# P02.183. Comparing the bioavailability of two forms of lipoic acid in multiple sclerosis

**DOI:** 10.1186/1472-6882-12-S1-P239

**Published:** 2012-06-12

**Authors:** S Salinthone, V Yadav, M Ganesh, G Cherala, L Shinto, D Koop, D Bourdette, D Carr

**Affiliations:** 1 Portland VA Medical Center, Portland, USA; 2 Oregon Health & Science University, Portland, USA

## Purpose

Lipoic acid (LA) stimulates production of an immunomodulatory molecule, cAMP, that may have therapeutic benefit in multiple sclerosis (MS). The study sought to determine the bioactivity of two forms of oral LA, racemic (R/S-LA) and R-LA, to identify factors that may improve the therapeutic effectiveness of LA in MS.

## Methods

Participants met the following criteria: age 18-70 years and a definite multiple sclerosis diagnosis. Consenting participants were randomized to 2 groups: R/S-LA (n=20) or R-LA (n=8). Blood was collected at baseline, 5, 10, 15, 30, 60, 90, 120, 180, 240, and 300 minutes after ingestion of a single 1200 mg LA dose. Bioactivity was determined by measuring immune cell cAMP levels (pmol/mg) at baseline and 240 minutes post ingestion. Group differences were analyzed by repeated measures analysis of variance (cAMP) and t-test (pharmacokinetics).

## Results

For R/S-LA, mean baseline cAMP levels were 8.6 (SE 0.83) pmol/mg protein, which increased to 14.1 (SE 1.7) 240 minutes post-ingestion. For R-LA, baseline cAMP levels were 5.7 (SE 0.54), which decreased to 4.7 (SE 0.41) 240 minutes post-ingestion. The mean change in cAMP levels were different between groups, p<0.01. The two groups showed no differences in serum LA levels at 240 minutes (p=0.29), but showed a difference in the mean AUC (min*mcg/ml): R/S-LA is 681.8 (SE 83.4) and R-LA is 389.5 (SE 43.9) (p<0.01). Tmax (minutes) between groups differed: R/S-LA was 81.0 (SE 8.9) and R-LA was 13.1 (SE 2.7); p<0.001.

## Conclusion

At 1200 mg oral dose and comparable serum LA levels at T240, R/S-LA showed higher cAMP levels compared to R-LA. Since R-LA had an earlier Tmax, it is plausible that increases in cAMP may have occurred at earlier undetected time-points. This pilot study warrants further investigation as differences between R-LA and R/S-LA in bioactivity and pharmacokinetics would impact clinical trial design and patient care.

